# Cobalt-induced oxidative stress and defense responses of *Adhatoda vasica* proliferated shoots

**DOI:** 10.1186/s12870-024-05915-7

**Published:** 2025-01-31

**Authors:** Abeer A. Radi, Fatma A. Farghaly, Fatma A. Al-Kahtany, Ahmed M. Zaher, Afaf M. Hamada

**Affiliations:** 1 Botany and Microbiology Department, Faculty of Science, Assiut University, Assiut, 71516 Egypt; 2 Biology Department, Faculty of Science, Ibb University, Ibb, Yemen; 3 Pharmacognosy Department, Faculty of Pharmacy, Assiut University, Assiut, 71515 Egypt; 4 Pharmacognosy Department, Faculty of Pharmacy, Tobruk University, Tobruk, Libya

**Keywords:** *Adhatoda vasica* L., Cobalt, Growth, Malondialdehyde, Antioxidants, FTIR analysis

## Abstract

**Background:**

Levels of heavy metal pollution are increasing due to industrial activities and urban expansion. While cobalt (Co) can be toxic to plants at high levels and isn’t considered essential, it plays a beneficial role in many enzymes and is critical for various biological functions. We conducted experiments to determine how *Adhatoda vasica* proliferated shoots react to exposure to various Co concentrations (50–1000 µM). We employed physiological and biochemical markers to elucidate the response mechanisms of this medicinal plant. The experiment was conducted in two replicates per treatment. The statistical analysis was based on data from four biological replicates per treatment.

**Results:**

Interestingly, the lowest Co concentration (50 µM) increased proliferated shoot growth by 41.45%. In contrast, higher Co concentrations (100–1000 µM) had detrimental effects on proliferated shoot development, water content, and photosynthetic pigment concentrations. As Co concentration increased, proliferated shoots produced excessive concentrations of reactive oxygen species (ROS). This ROS overproduction is believed to be the primary cause of oxidative damage, as evidenced by the elevated concentrations (18.46%-72.84%) of malondialdehyde (MDA) detected. In response to Co stress, non-enzymatic antioxidants were activated in a concentration-dependent manner. Co administration significantly increased the concentrations of different stress-protective compounds in shoots, including total antioxidants (133.18%), ascorbic acids (217.94%), free and bound phenolics (97.70% and 69.72%, respectively), proline (218.59%), free amino acids (206.96%), soluble proteins (65.97%), and soluble carbohydrates (18.52%). FTIR analysis further corroborated changes in the chemical composition of proliferated shoots. The analysis revealed variations in the peaks associated with major macromolecules, including phenolic compounds, lipids, proteins, carbohydrates, cellulose, hemicellulose, and sugars.

**Conclusions:**

Our study offers the first comprehensive investigation into mechanisms by which Co stress triggers oxidative damage and alters functional groups in the medicinal plant, *Adhatoda vasica*.

**Supplementary Information:**

The online version contains supplementary material available at 10.1186/s12870-024-05915-7.

## Background

Heavy metals (HMs) naturally occur worldwide, but human activities like mining, agriculture, and improper waste disposal from sewage and industry significantly increase their presence in the environment [1]. Deep within the Earth’s crust, HMs exist as a non-degradable component of the planet’s geological structure [2]. The excessive extraction of natural resources has facilitated the release of HMs from the Earth’s crust. While some heavy metals occur naturally in soil, human activities are a major contributor to their increased presence [3]. Studies have shown that human activities contribute to the atmospheric release of HMs at a rate that is approximately three times higher than natural processes [4]. HMs are discharged into soil and water bodies in both dissolved and solid forms. In water bodies, they settle on the seafloor or adhere to solid objects [5]. In soil, HMs tend to accumulate due to their resistance to microbial degradation, as opposed to organic contamination [6]. As a result, they persist in the soil for extended periods. HMs pose a significant threat to the environment. They can hinder the natural breakdown of organic matter, contaminate groundwater, harm soil fauna, and disrupt microbial communities [7]. These impacts can have long-lasting consequences for ecosystems and human health [8]. HMs pose a significant health risk due to their non-biodegradable nature and prolonged retention in biological systems [9]. HMs are essential for plant growth at low levels but become toxic at higher concentrations [10]. Plant toxicity from HMs varies based on factors like plant species, metal form, soil type, and pH [10].

Cobalt (Co) is essential for the development of lower plants and animals, including humans and some mammals, but its role in higher plants is less clear [11]. While it is a beneficial nutrient in small doses, most higher plants do not consider it essential. It helps plants grow better by regulating water use and reducing loss [12]. According to Pilon-Smits et al. [13], it also prevents ethylene production, which delays the senescence of leaves. While Co is essential for plant growth, high levels can be toxic [14]. High levels of Co can severely damage plant cells, including key structures like the photosynthetic apparatus, which is essential for energy production, and the cell membrane, which controls what enters and leaves the cell [15, 16]. Co exposure can induce toxic impacts on photosynthetic machinery, gas exchange, and antioxidant systems [17, 18]. These effects lead to reduced plant growth and chlorophyll concentration. Within plant cells, Co exposure can trigger Fenton reactions, leading to increased production of reactive oxygen species (ROS) [17]. This rise in ROS can damage biomolecules via DNA degradation, lipid peroxidation, or enzyme inactivation [19]. In contrast, plants can develop adaptive responses to mitigate the toxic effects of metals. These responses include scavenging ROS or compartmentalizing metals in fewer sensitive organs like vacuoles and cell walls [20]. Furthermore, some metabolites play a crucial role in scavenging ROS during plant exposure to heavy metals [21–23].

*Adhatoda vasica* L. (*Justicia adhatoda* L.) is a small evergreen shrub native to tropical and subtropical regions. *A. vasica* is a highly regarded medicinal plant with a long history of use in Ayurvedic and Unani medicine. In Egypt, *Justicia adhatoda* (*A. vasica*) is mostly grown for its therapeutic qualities [24]. Although rare, it may be a good hedge plant in fields and gardens [25]. *A. vasica*, a prominent medicinal plant native to Asia, belongs to the Acanthaceae family. It is well-known in traditional medicine and has a long history of use for various health conditions. These include respiratory ailments, radiomodulation, blood sugar control, cardiovascular health, tuberculosis, viral infections, liver protection, protection against cell damage, and antioxidant properties [26–28]. Its extensive use in traditional medicine led to its inclusion in the World Health Organization's (WHO) manual, “The Use of Traditional Medicine in Primary Health Care.” This manual is a resource for healthcare workers in Southeast Asia, informing them about the potential benefits of local plants [29].

Maintaining high-quality herbal products is essential. Herbal preparations may contain contaminants like heavy metals or pesticide residues. Environmental factors, including air and soil pollution, along with harvesting and handling practices, significantly contribute to the contamination of herbal plants [30]. While essential in small amounts, heavy metals can be toxic to both humans and herbal plants at high concentrations.

Despite extensive research on cobalt’s toxicity, a significant knowledge gap persists regarding its precise impact on the functional groups of *A. vasica*, hindering our understanding of its potential ecological and health implications. FTIR analysis identified macromolecules as key components in the chelation and sequestration of HMs [31]. The impact of Co treatments on FTIR functional groups has not been previously reported, suggesting a promising avenue for future research. This research investigates the effects of increasing cobalt concentrations (50–1000 µM) on the growth, non-enzymatic antioxidants, and FTIR functional groups of *A. vasica*. This study provides a comprehensive analysis of Co toxicity in *A. vasica* proliferated shoots. This study assesses *A. vasica*’s tolerance to Co toxicity and its suitability for cultivation in Co-contaminated areas.

## Results

### Percent of damage

**Fig. 1:**
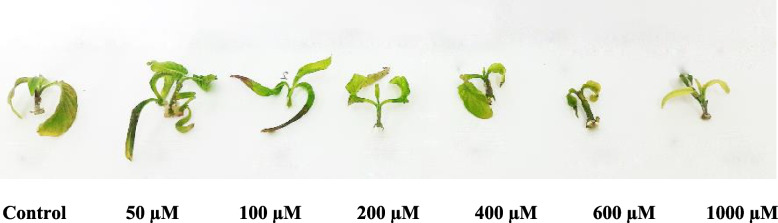
Effect of cobalt concentrations (0–1000 µM) on *Adhatoda vasica* shoot proliferation over 30 days

**Fig. 2:**
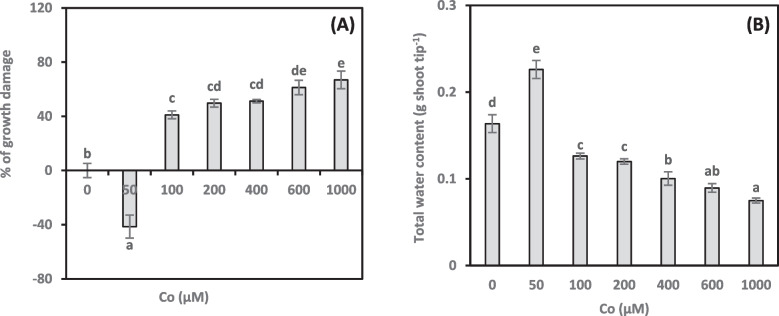
% of growth damage (**A**) and total water content (**B**) of *Adhatoda vasica* proliferated shoots under the influence of different concentrations of Co for 30 days. The data are means ± SD (*n* = 10). The letters indicate statistically significant differences (*P* ≤ 0.05)

We measured *A. vasica* proliferated shoot growth damage to assess the impact of different Co concentrations (Figs. 1, 2A). The current study revealed that at a low concentration of Co (50 µM), *A. vasica* proliferated shoot growth increased by 41.5% compared to the control. However, shoot growth was decreased at higher concentrations of Co (100–1000 µM). Compared to the control shoots, high cobalt concentrations (100–1000 µM) decreased growth by 41.07%, 49.65%, 51.14%, 61.23%, and 66.66%, respectively.

### Total water content

TWC in proliferated shoots is a critical physiological factor influencing plant growth. Therefore, we examined how Co treatments impacted TWC (Fig. 2B). Our results revealed that a low concentration of Co (50 µM) significantly increased the TWC of shoots (38.21%) over the control group. However, with increasing Co concentrations, the TWC progressively decreased. The highest Co concentration (1000 µM) treatment lowered the TWC in shoots by 54.16% compared to the control group. Furthermore, a significant negative correlation (−0.971**, online Supplementary Table 1) was found between the TWC and growth damage in Co-treated plants.

### Photosynthetic pigments

**Fig. 3:**
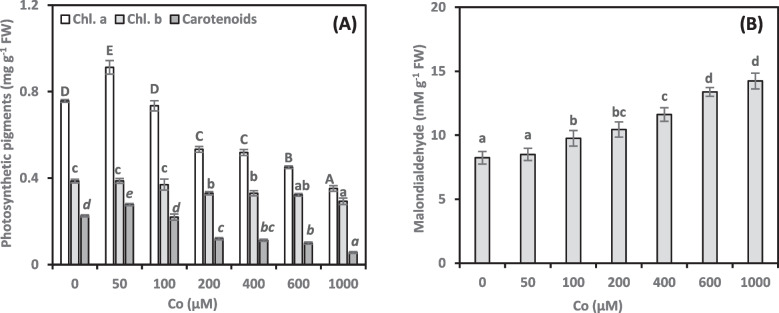
Photosynthetic pigments [chlorophyll *a* (Chl. a), chlorophyll *b* (Chl. b), and carotenoids; (**A**)] and malondialdehyde [MDA; (**B**)] content of *Adhatoda vasica* proliferated shoots under the influence of different concentrations of Co for 30 days. The data are means ± SD (*n* = 4). The letters, uppercase for chl. *a*, lowercase small for chl. *b* and MDA, and italicized for carotenoids, indicate statistically significant differences (*P* ≤ 0.05)

Co exposure significantly impacted the concentration of chl. *a*, *b*, and carotenoid pigments (Fig. 3A). A low concentration of Co (50 µM) treatment increased chl. *a* and carotenoid pigments by 20.47% and 23.12%, respectively. However, chl. *b* concentration remained unchanged. Conversely, exposure to 100 µM Co concentrations did not affect photosynthetic pigments. Plants exposed to Co at 200–1000 µM showed a gradual reduction in photosynthetic pigments. Notably, 1000 µM Co caused significant decreases in chl. *a* (53.49%), chl. *b* (24.18%), and carotenoids (75.17%). Moreover, in Co-treated plants, there were negative associations (−0.900**, −0.789**, and −0.886**, respectively; online Supplementary Table 1) between growth damage and chl. *a*, *b*, and carotenoids.

### Malondialdehyde

Our results showed that when Co concentration increased, MDA generation increased in a concentration-dependent manner (Fig. 3B). The shoots exposed to the highest Co concentration (1000 µM) exhibited the most significant increase in MDA levels (72.84%) compared to control shoots. Conversely, the lowest Co concentration (50 µM) did not significantly increase the MDA concentrations compared to the control. Moreover, Co treatments induced a strong positive correlation between growth damage and MDA concentrations (0.784**, online Supplementary Table 1).

### Total antioxidants

**Fig. 4:**
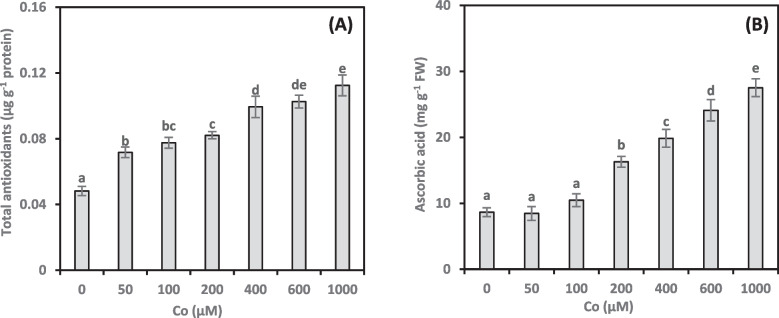
Total antioxidants (**A**) and ascorbic acid (**B**) content of *Adhatoda vasica* proliferated shoots under the influence of different concentrations of Co for 30 days. The data are means ± SD (*n* = 4). The letters indicate statistically significant differences (*P* ≤ 0.05)

We measured the total antioxidant concentrations in the shoots of *A. vasica* to evaluate ROS scavenging during Co stress (Fig. 4A). Antioxidant levels rose steadily as Co concentrations in the medium increased. Compared to the control, Co concentrations of 50, 100, 200, 400, 600, and 1000 µM resulted in increases of 48.79%, 60.72%, 70.24%, 105.97%, 112.73%, and 133.18%, respectively. Additionally, we found a significant positive correlation (0.716**, online Supplementary Table 1) between growth damage and total antioxidant concentrations due to Co treatments.

### Ascorbic acid

To investigate the role of AsA in mitigating Co stress, we measured AsA concentrations in Co-treated *A. vasica* shoots (Fig. 4B). Low Co concentrations (50 and 100 µM) had minimal effect on AsA concentrations in shoots. However, higher Co concentrations (200–1000 µM) significantly increased the AsA concentrations. Compared to the control, Co treatments enhanced AsA by 88.20%, 129.28%, 178.33%, and 217.94% at 200, 400, 600, and 1000 µM, respectively. Notably, there was a significant positive correlation (0.783**, online Supplementary Table 1) between growth damage and AsA concentrations in Co-treated shoots.

### Phenolic compounds

**Fig. 5:**
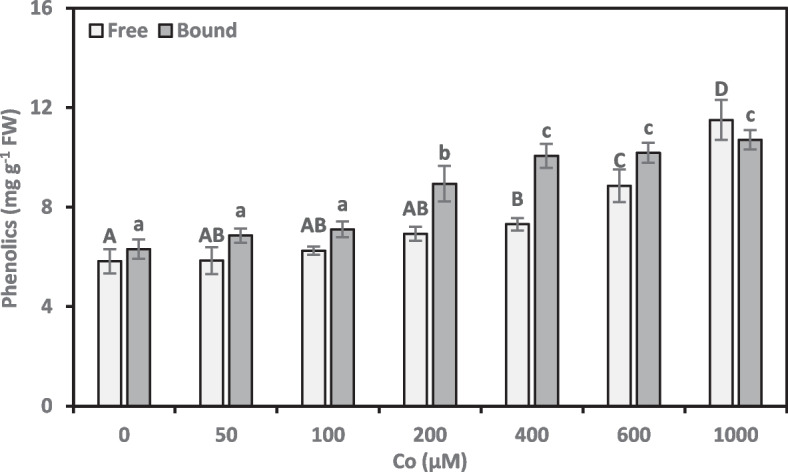
Phenolic compounds (free and bound) content of *Adhatoda vasica* proliferated shoots under the influence of different concentrations of Co for 30 days. The data are means ± SD (*n* = 4). The letters, uppercase for free and lowercase for bound phenolics, indicate statistically significant differences (*P* ≤ 0.05)

We investigated the effects of Co on free and bound phenolic synthesis in *A. vasica* shoots (Fig. 5). For low Co concentrations of 50–200 µM, there was no discernible increase in free phenolic concentrations above the control. However, higher Co concentrations (400–1000 µM) significantly increased free phenolics. Only treatments with 400, 600, and 1000 µM Co resulted in significant increases of 25.55%, 52.17%, and 97.70% in free phenolics, respectively, compared to the control. Furthermore, Co treatments resulted in a strong positive correlation (0.638**) between growth damage and free phenolic concentrations.

Based on the results in Fig. 5, low Co concentrations of 50 and 100 µM did not significantly elevate bound phenolic concentrations in shoots. Conversely, higher Co concentrations (200–1000 µM) significantly stimulated bound phenolic production. These higher Co treatments caused substantial increases in the bound phenolics, ranging from 41.78% to 69.72% compared to the control. We also found a significant positive correlation (0.776**, online Supplementary Table 1) between growth damage and bound phenolic concentrations due to Co treatments.

### Proline

**Fig. 6:**
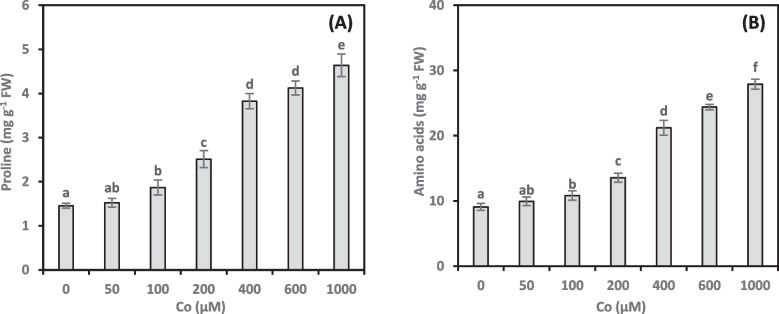
Proline (**A**) and other free amino acids (**B**) content of *Adhatoda vasica* proliferated shoots under the influence of different concentrations of Co for 30 days. The data are means ± SD (*n* = 4). The letters indicate statistically significant differences (*P* ≤ 0.05)

Proline levels in *A. vasica* shoots were examined to gauge cobalt’s effect on osmoprotectant production (Fig. 6A). Proline concentrations were significantly upregulated by Co treatments in a dose-dependent manner. Compared to untreated controls, Co treatments (100–1000 µM) led to a substantial increase in proline levels, ranging from 28.22% to 218.59%. In addition, a strong positive correlation (0.772**, online Supplementary Table 1) existed between proline concentration and Co-induced growth damage.

### Amino acids

Co treatments raised the concentrations of amino acids in *A. vasica* shoots (Fig. 6B). The highest Co concentration (1000 µM) resulted in a substantial increase of 206.96% in amino acid concentrations compared to the control. However, the lowest Co concentration (50 µM) did not significantly elevate amino acid concentrations compared to the control. Furthermore, Co treatments caused significantly positive correlations between growth damage and amino acid concentrations (0.729**, online Supplementary Table 1).

### Soluble proteins

**Fig. 7:**
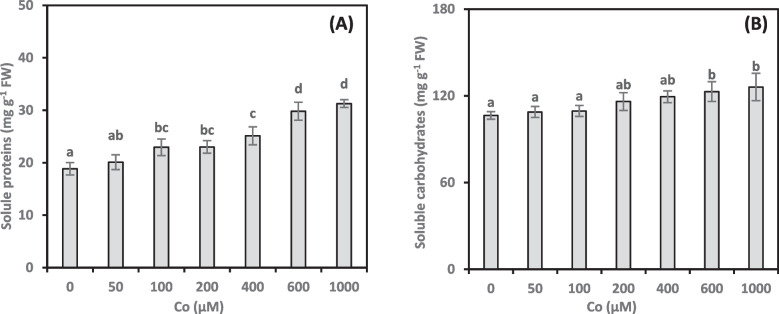
Soluble proteins (**A**) and soluble carbohydrates (**B**) content of *Adhatoda vasica* proliferated shoots under the influence of different concentrations of Co for 30 days. The data are means ± SD (*n* = 4). The letters indicate statistically significant differences (*P* ≤ 0.05)

Soluble proteins are critical for regulating plant growth and development during stress responses. Therefore, we examined the effect of Co on soluble proteins in *A. vasica* shoots (Fig. 7A). Dose-dependently, Co treatments increased the synthesis of soluble proteins. Compared to the control, protein concentrations increased progressively from 21.77% to 65.97% with increasing Co concentrations (100–1000 µM). Interestingly, the lowest Co concentration (50 µM) did not significantly enhance protein concentrations. Moreover, Co treatments induced a strong positive correlation (0.753**, online Supplementary Table 1) between soluble proteins and growth damage.

### Soluble carbohydrates

Soluble carbohydrates are crucial in maintaining cell turgor and preventing dehydration under stress. In this study, we investigated the effects of Co on soluble carbohydrate concentrations in *A. vasica* shoots (Fig. 7B). Our results showed that low and moderate Co concentrations (50–400 µM) did not significantly affect soluble carbohydrate concentrations compared to the control. However, higher Co treatments (600 and 1000 µM) significantly increased carbohydrate concentrations by 15.52% and 18.52%, respectively. Additionally, we observed a strong positive correlation (0.630**, online Supplementary Table 1) between soluble carbohydrate concentration and Co-induced growth damage.

### Fourier transform infrared analysis

**Fig. 8:**
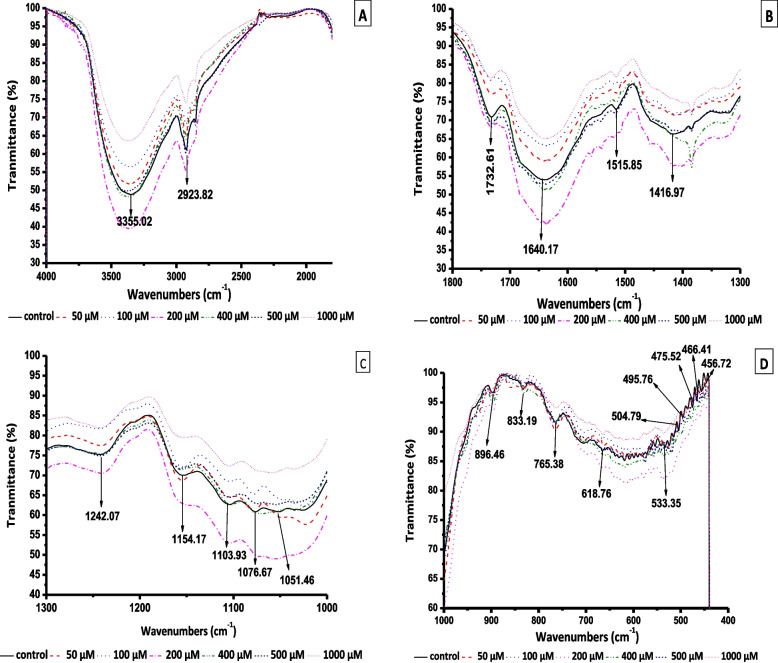
Fourier-transform infrared spectroscopy (FTIR) spectra [(**A**) range 4000–2000 cm^−1^, (**B**) range 1800–1300 cm^−1^, (**C**) range 1300–1000 cm^−1^, and (**D**) range 1000–400 cm^−1^] of *Adhatoda vasica* proliferated shoots under the influence of different concentrations of Co for 30 days

This study investigated the effects of Co on functional groups in the *A. vasica* proliferate shoots (Fig. 8A-D, Table 1). Treatments with 100 and 200 µM Co caused the formation of two weak peaks at 3852.91 cm^−1^ and 3853.09 cm^−1^, respectively. At 50, 100, 200, or 1000 µM of Co, there were no appreciable changes in transmission for the broad peak at 3355.02 cm^−1^ (control). However, 400 and 600 µM Co concentrations caused a positive shift in this peak by 7.42 cm^−1^ and 27.08 cm^−1^, respectively.

**Table 1:** Fourier-transform infrared spectroscopy (FTIR) spectra (range 4000–400 cm^−1^) of *Adhatoda vasica* proliferated shoots under the influence of different concentrations of cobalt for 30 days.* A. vasica* shows 27 characteristic peaks in its FTIR spectrum

Number	Frequency (cm^−1^)						
	**Cobalt (µM)**						
	**0**	**50**	**100**	**200**	**400**	**600**	**1000**
**1**			**3852.91**	**3853.09**			
**2**	**3355.02**	**3354.67**	**3355.12**	**3355.73**	**3362.44**	**3382.1**	**3354.55**
**3**	**2923.82**	**2926.23**	**2924.53**	**2926.17**	**2926.83**	**2924.86**	**2923.67**
**4**						**2853.37**	
**5**	**1732.61**	**1730.55**		**1733.5**	**1730.17**	**1733.15**	**1733.01**
**6**	**1640.17**	**1640.99**	**1635.76**	**1636.15**	**1640.24**	**1636.2**	**1640.01**
**7**	**1515.85**	**1515.48**		**1559.09**			**1515.59**
**8**	**1416.97**	**1413.86**	**1418.34**	**1404.68**		**1400.18**	
**9**			**1384.88**		**1384.44**	**1383.88**	**1384.53**
**10**	**1242.07**	**1240.75**	**1246.23**	**1240.33**	**1241.65**	**1242.08**	**1241.62**
**11**	**1154.17**	**1155.08**	**1154.31**			**1157**	
**12**	**1103.93**						**1103.85**
**13**	**1076.67**	**1078.18**	**1077.81**		**1069.52**		**1060.65**
**14**	**1051.46**	**1022.7**	**1023.44**	**1054.41**		**1054.52**	
**15**	**896.46**			**896.54**	**896.86**	**896.94**	**896.34**
**16**	**833.19**	**833.89**	**854.38**	**833.05**			**832.07**
**17**	**765.38**	**765.67**	**756.46**	**765.06**		**765.05**	**764.94**
**18**		**705.28**					
**19**	**618.76**	**617.06**		**617.47**	**618.68**		**619.14**
**20**		**576.9**	**577.86**			**575.57**	
**21**						**556.92**	
**22**	**533.35**	**525.69**	**530.96**	**538.71**	**536.82**	**542.82**	**537.14**
**23**	**504.79**				**512.96**	**519.19**	
**24**	**495.76**						
**25**	**475.52**		**469.77**				
**26**	**466.41**	**460.09**	**458.15**			**462.32**	
**27**	**456.72**						**449.16**

FTIR analysis indicated that Co treatments did not affect the peak at 2923.82 cm^−1^ in *A. vasica* shoots (Fig. 8A, Table 1). After plants were exposed to 600 µM Co, FTIR analysis identified a new spectral peak at 2853.37 cm^−1^. FTIR analysis revealed that Co treatments had minimal influence on the 1732.61 cm^−1^ peak, except at 100 µM, where it was completely suppressed (Fig. 8B, Table 1). The peak at 1640 cm^−1^ was largely unaffected by Co treatments; however, concentrations of 100 and 200 µM led to slight decreases in transmission by 4.41 cm^−1^ and 4.02 cm^−1^, respectively.

The 1515.85 cm^−1^ peak displayed varying responses to Co treatments (Fig. 8B, Table 1). It increased at 200 µM but disappeared at 100, 400, and 600 µM, while 50 and 1000 µM had minimal effects. Co treatments had varying effects on the small peak at 1416.97 cm^−1^; low concentrations (50 and 100 µM) did not significantly alter the peak transmission. However, higher Co treatments at 200 and 600 µM induced a decrease in transmission by 12.29 cm^−1^ and 16.79 cm^−1^, respectively. Interestingly, the peak disappeared entirely at 400 and 1000 µM Co concentrations. Co treatments caused additional peaks to emerge; treatments at 100, 400, 600, and 1000 µM resulted in new peaks at 1384.88 cm^−1^, 1384.44 cm^−1^, 1383.88 cm^−1^, and 1384.53 cm^−1^, respectively (Fig. 8B, Table 1).

The 1242.07 cm^−1^ peak was largely unaffected by Co, except for a slight increase of 4.16 cm^−1^ at 100 µM (Fig. 8C, Table 1). Treatment with Co showed different results on the 1154.17 cm^−1^ peak; the concentrations of 50, 100, and 600 µM Co did not significantly alter it. However, the peak disappeared entirely at concentrations of 200, 400, and 1000 µM Co (Fig. 8C, Table 1). Low cobalt levels (50–600 µM) eliminated the small peak at 1103.93 cm^-1^, while higher concentrations (1000 µM) had minimal effect (Fig. 8C, Table 1). Treatments with low Co concentrations (50 and 100 µM) did not significantly alter the peak at 1076.67 cm^−1^, while 400 and 1000 µM caused a reduction in the transmission by 7.15 cm^−1^ and 16.02 cm^−1^. However, the peak disappeared entirely at concentrations of 200 and 600 µM Co (Fig. 8C, Table 1). Co exhibited distinct effects on the small peak at 1051.46 cm^−1^ (control); low Co concentrations (50 and 100 µM) decreased its transmission by 28.76 cm^−1^ and 28.02 cm^−1^, respectively. In contrast, Co concentration at 200 µM did not significantly affect the peak transmission, while it disappeared entirely at 400 and 1000 µM.

Low Co concentrations (50 and 100 µM) masked the peak at 896.46 cm^−1^, while higher concentrations (200–1000 µM) did not significantly affect the peak transmission (Fig. 8D and Table 1). The effects of Co treatment differed on the peak at 833.19 cm^−1^; 50, 200, and 1000 µM Co did not significantly affect its transmission. In contrast, 100 µM Co increased transmission by 21.19 cm^−1^, while 400 and 600 µM Co masked the peak (Fig. 8D, Table 1). Co treatment effects on the small peak at 765.38 cm^−1^ were inconsistent; concentrations of 50, 200, 600, and 1000 µM did not significantly affect it, whereas 100 µM decreased its transmission by 8.92 cm^−1^. However, 400 µM Co masked the peak entirely (Fig. 8D, Table 1). At 50 µM, Co created a new, tiny peak at 705.28 cm^−1^. Co concentrations of 50, 200, 400, and 1000 µM did not affect the transmittance of the peak at 618.76 cm^−1^. Conversely, the peak disappeared entirely following Co treatment at 100 and 600 µM. Co at 50, 100, and 600 µM concentrations resulted in new peaks at 576.90 cm^−1^, 577.86 cm^−1^, and 575.57 cm^−1^, respectively (Fig. 8D, Table 1). The effects of Co treatment varied on the peak at 533.35 cm^−1^; the lowest concentration (50 µM) significantly decreased the peak transmission (7.66 cm^−1^). In contrast, 200 and 600 µM increased transmission by 5.36 cm^−1^ and 9.47 cm^−1^, while 100 and 400 µM Co did not significantly increase it, respectively (Fig. 8D, Table 1). Co treatments at 400 and 600 µM increased the small peak 504.79 cm^−1^ transmission (8.17 cm^−1^ and 14.4 cm^−1^, respectively), but 50, 100, 200, and 1000 µM levels suppressed it (Fig. 8D and Table 1). With Co treatments, the small peak at 495.76 cm^-1^ disappeared (Fig. 8D, Table 1). Co treatments masked the 475.52 cm^−1^ peak, except for 100 µM Co, which decreased it by 5.75 cm^−1^. Co 50, 100, and 600 µM levels decreased the 466.41 cm^−1^ peak transmission by 6.32 cm^−1^, 8.26 cm^−1^, and 4.09 cm^−1^, respectively. Co treatments masked the peak at 456.72 cm^−1^, except for 1000 µM Co, which decreased it by 7.56 cm^−1^.

FTIR analysis revealed a decrease in most peak intensities following Co treatment. Interestingly, the 200 µM concentration exhibited a distinct trend, increasing the intensity of most peaks.

## Discussion

This study investigates the effects of varying Co concentrations on medicinal plant *A. vasica* using a novel and comprehensive approach. We examine plant growth, non-enzymatic antioxidants, and the functional groups within their biomolecules.

For optimal development, plants need not just essential minerals but also elements that provide additional benefits. This requirement varies across different plant species. Co, a crucial trace element for plants in small amounts, becomes toxic at high concentrations. Our study found that low concentrations of Co enhanced shoot growth, suggesting a beneficial role in plant development. Co is essential for interactions between nickel, iron, and zinc, contributing to cell equilibrium [11]. However, high Co concentrations resulted in stunted growth, likely due to the accumulation of Co ions within the plants, which may have disrupted essential processes. Co toxicity has a similar deleterious effect on wheat growth, as Zahid et al. [32] demonstrated. This growth damage is associated with numerous factors, including reduced cell division [33], oxidative stress, inhibited photosynthesis, and iron deficiency [34]. In addition, impaired nutrient uptake, plant water status, and stomatal conductance [35] contribute to these detrimental effects. The significant negative correlation between total water content and shoot growth damage supports the theory that low water content causes growth loss. Conversely, our experiments showed that low Co concentrations boosted water content in proliferated shoots, indicating a beneficial role.

Chlorophyll levels are a reliable indicator of plant health and stress tolerance, reflecting their ability to photosynthesize effectively [36]. HM stress can damage photosynthetic pigments, which disrupt the electron supply chain and cause an excess of ROS [37, 38]. Chloroplasts are among the main organelles harmed by Co toxicity [32]. High Co concentrations cause photosynthetic pigment deficiencies, likely due to chloroplast dysfunction (Fig. 3A). The presence of Co ions in chloroplasts disrupts chlorophyll production, competing with essential elements like Mg or Fe. This competition leads to a decrease in overall chlorophyll concentrations [39]. According to Sree et al. [16], Co inhibits enzymes responsible for synthesizing chlorophyll precursors. Thylakoid damage and/or oxidation of chloroplast membranes might explain pigment deficiencies [32]. Our findings on Co toxicity align with observations in duckweed and wheat [16, 32]. These studies, like ours, reveal various detrimental effects of Co on photosynthesis, including reduced photosynthetic pigment concentrations, CO_2_ assimilation, photosystem II performance, and electron transport rate. Our results demonstrate that pigment concentrations play a critical role in driving plant growth. Plants exposed to lower Co concentrations displayed higher photosynthetic pigment concentrations, suggesting their chloroplasts were less susceptible to cobalt’s detrimental effects. This aligns with our observation of a strong link between pigment concentrations and plant growth, highlighting their vital role. Supporting this notion, Zeid [33] reported significant increases in the photosynthetic pigments of bean plants under low Co exposure. The author attributed this rise in pigments to potentially enhanced magnesium transport.

Exposure to heavy metals triggers increased production of ROS in plants, disrupting the cellular redox balance and leading to oxidative stress [40]. Co toxicity may cause the development of ROS, which can alter membranes and damage metabolic processes [32]. Increased MDA concentrations reveal Co-induced membrane damage, with severity increasing alongside Co concentrations (Fig. 3B). Co exposure can elevate ROS levels, impairing electron transport within plastids and mitochondria. Co exposure at high concentrations induces oxidative stress, ultimately suppressing growth. This finding demonstrates that Co exposure significantly disrupts membrane integrity. Our finding of a strong positive correlation between MDA concentration and growth damage aligns with recent work by Zahid et al. [32]. The authors demonstrated that Co exposure triggers oxidative stress in wheat, leading to cellular damage in organelles and disruption of membrane integrity.

The higher MDA concentrations in the samples indicate that the Co treatments caused oxidative stress. This biochemical marker indicates lipid peroxidation, a hallmark of oxidative damage. While HMs trigger responses in plants’ antioxidant systems, the effect (activation or suppression) depends on the tissue type, plant species, specific metal, and severity of stress [41, 42]. To investigate *A. vasica*’s antioxidant response to Co stress, we determined the total antioxidant concentrations in its shoots (Fig. 4A). *A. vasica* shoots exhibited a dose-dependent increase in total antioxidants in response to increasing Co concentrations, suggesting a protective mechanism against oxidative stress. Proliferated *A. vasica* shoots demonstrate elevated antioxidant levels, indicating their potential to protect against Co-induced oxidative stress. However, the positive correlation between total antioxidants and growth damage indicates a more complex relationship, requiring further investigation. The impact of HMs on total antioxidant levels has been extensively examined [22], but the precise effects of Co on these antioxidant levels have not been well studied.

AsA plays a multifaceted role in plants. Additionally, it helps maintain redox balance within the plant. Following Co treatments, ascorbate concentrations increased (Fig. 4B), which suggests that AsA is actively involved in reducing intracellular oxidative stress. Our finding of a positive correlation between AsA and growth damage aligns with Radi et al. [22]. The authors showed that high levels of cellular oxidants can trigger increased AsA synthesis. These findings are consistent with observations in wheat varieties exposed to Co stress [32]. While AsA can offer some protection against Co-induced damage, its effectiveness is limited, and it cannot fully counteract the harm. Further investigation is needed to elucidate the precise mechanisms by which AsA interacts with Co to influence plant health.

Phenolic compounds act as antioxidants, scavenging ROS generated by (a)biotic stresses. According to Giada [43], phenolic compounds can be classified into soluble and insoluble groups based on their chemical structure and location within the plant. Soluble phenolics comprise a diverse group of low-molecular-weight compounds, including free simple phenolics and other unbound phenolics. Bound phenolics are typically ester-linked to cell wall polysaccharides and can be modified in response to stress. The study revealed a significant increase in free and bound phenolics within the shoots of plants exposed to cobalt stress (Fig. 5), suggesting an adaptive strategy. The correlations between growth damage and free, bound phenolics indicate that plants under stress, like Co, can direct their resources into defensive mechanisms rather than growth. In agreement with our results, Zahid et al. [32] also observed a significant increase in total phenolic concentration in wheat plants exposed to Co stress. Co treatment resulted in a significant increase in the production of bound phenolics in plants. The strong positive correlation between bound phenolics and growth damage supports the findings of Fan et al. [44]. In maize, the authors found a correlation between the accumulation of bound phenolics and the inhibition of cell wall expansion and root growth caused by water deficiency. Our study also revealed that Co stress significantly reduced the total water content of proliferated shoots. The interplay between Co stress and phenolic metabolism warrants further exploration.

Proline’s stress-protective functions in plants include metal chelation, which helps prevent oxidative damage, and molecular chaperoning, which aids in protein folding and stability during adverse conditions. Proline acts as an antioxidant, scavenging reactive ROS to protect cells from oxidative damage. Proline also activates specific genes essential for plant stress recovery [45, 46]. Our study demonstrated an increase in proline concentration alongside rising Co exposure, suggesting that proline synthesis is upregulated as an adaptive strategy to mitigate Co-induced toxicity. Proline’s positive correlation with growth damage supports its role in stress response. However, proline can only partially undo the harm that Co has produced; it cannot completely reverse it [47]. Our findings align with recent studies [32, 48] showing proline accumulation in plants under Zn and Co stress. This accumulation likely counteracts the membrane damage and lipid peroxidation caused by Co stress [49]. Under metal stress, plants accumulate proline, a versatile molecule with multiple protective functions [50, 51]. Consistent with our results, Ali et al. [38] observed a significant increase in proline concentration in *Brassica napus* plants exposed to Co stress. This finding strengthens the evidence for proline accumulation as a response mechanism to Co stress.

In response to HM stress, plants synthesize amino acids that can trap and chelate metal ions, contributing to their tolerance of these toxins [52]. Amino acids serve as building blocks for more than just proteins. They also contribute to the biosynthesis of secondary metabolites, such as lipids and carbohydrates [53, 54]. The findings of this study demonstrate a positive correlation between Co supply and amino acid concentrations, indicating that Co may play a regulatory role in amino acid biosynthesis pathways. Lwalaba et al. [23] reported an increase in the concentration of most amino acids in barley roots exposed to Co or copper, suggesting that these metals may influence amino acid metabolism. The positive correlation between amino acid concentrations and growth damage supports their involvement in the plant's stress response. Abdel-Wahab et al. [31] reported similar findings in *Solanum nigrum* callus treated with ZnO nanoparticles. The authors suggested a potential role for these amino acids in enhancing Zn chelation.

Plants use soluble proteins to defend against HM stress. These proteins act as crucial regulators, ensuring continued growth and development. Higher Co levels led to increased soluble protein concentrations in *A. vasica* shoots. However, the increase in soluble protein concentrations did not prevent the Co-induced growth damage. Previous studies have shown that Co stress triggers metalloproteinase gene expression and significantly increases its accumulation in *Brassica napus* plants [17]. Plants exposed to Co show increased metalloproteinase gene expression, suggesting a protective role in mitigating the oxidative damage caused by this metal. A deeper understanding of the complex interaction between Co stress and soluble protein metabolism is crucial for future research.

Carbohydrates are essential biomolecules for plants and animals, playing crucial roles in energy storage and cellular processes through metabolism. Exposure to abiotic factors like HMs triggers changes in plant cells, including adjustments to non-structural carbohydrate concentrations [55]. This study revealed a significant increase in soluble carbohydrate concentrations in *A. vasica* shoots when exposed to high Co concentrations. This increase in soluble carbohydrates may improve osmotic potential in the shoots, allowing plants to tolerate the toxic effects of Co. A strong correlation between soluble carbohydrates and growth damage suggests that plants may employ alternative strategies to cope with Co toxicity. Under HM stress, plants often activate enzymes involved in sucrose metabolism, leading to increased soluble carbohydrate production [56]. According to Abdel-Wahab et al. [31], the capacity of *Solanum nigrum* plants to create osmolytes and control stress may be the cause of their higher soluble carbohydrate content. These processes are essential for the plant’s growth and development. Further exploration of how Co stress modulates soluble carbohydrates metabolism could reveal key mechanisms underlying plant responses to HMs.

FTIR spectroscopy offers a powerful approach for investigating the chemical composition and functional groups of biological substances [57]. Detecting specific functional groups and chemical bonds provides valuable information about the types of biochemical compounds present in the sample [57]. FTIR spectra of *A. vasica* shoots provided valuable information about the molecular-level effects of Co stress on plant tissue. Türker-Kaya and Huck [58] assigned the peak area at 3355.02 cm^−1^ to N–H and O–H functional groups in biomolecules such as proteins, carbohydrates, phenolics, and alcohols. This peak area did not alter at low Co concentrations, but at high concentrations, it exhibited a noticeable expansion. Although 1000 µM Co did not alter the peak area, the intensity decreased. The reduction in peak intensity at high Co concentrations suggests a potential alteration in the binding patterns between molecules. A peak shift suggests changes in the composition of alcohols, phenolics, proteins, and carbohydrates. The observed changes may be due to increased Co chelation, potentially altering these biomolecules’ composition. Similar peak modifications were noted by Radi et al. [22] in their investigation of ZnO nanoparticle impacts on pomegranate callus. The authors suggested that there might have been an increase in Zn chelation.

The peak at 2950–2845 cm^-1^, assigned to -CH_2_ groups, primarily originates from lipids with minimal contributions from DNA, proteins, and carbohydrates [58]. These macromolecules are the lipid bilayer’s building blocks, forming the cell membrane [59]. The lower intensity of lipid-related peaks indicates increased lipid oxidation under Co stress. As previously demonstrated, the elevated MDA content aligns with these observations [60].

Under Co stress, certain protein peaks exhibited reduced absorption or complete disappearance, while others showed increased absorption. This indicates a potential shift in protein populations or altered conformations in the cell. According to Dumas and Miller [61], peaks in the 1700–1600 cm^−1^ region represent amide I carbonyl groups in proteins. The investigation found that the amide I peak transmission exhibited little shift at low Co concentrations. This shift suggests potential conformational changes within proliferated shoot proteins. Co stress decreased the intensity of these peaks, indicating a possible reduction in protein abundance or altered protein structure. Bandekar [62] reported that peaks in the 1555–1309 cm^−1^ region correspond to amides II and III of proteins. Analysis of protein peak profiles showed various alterations following Co treatments, suggesting conformational shifts in proliferated shoot proteins. These intriguing results warrant further investigation for confirmation.

Peaks in the 1250–1100 cm^−1^ region suggest the presence of various biomolecules, including pectins, lignin, suberin, cutin, xylan, hemicellulose, cellulose, and amide IV (protein) [58]. Co treatments generally did not affect the peak at 1242.07 cm^−1^, and high concentrations reduced its intensity. This finding could suggest a change in lignin with perhaps fewer hydrogen bonds. The changes in lignin content may be associated with reduced cell wall expansion and increased stiffness. These factors may be responsible for reduced plant growth, although further research is required for definitive confirmation.

Peaks between 1072 cm^−1^ and 1040 cm^−1^ indicate CO stretching in hemicellulose and cellulose [63]. Reduced peak areas in these regions indicate a potential decrease in the synthesis of cellulose, hemicellulose, and related carbohydrates involved in cell wall formation. High concentrations of Co treatments decreased the intensity of these peaks. This result suggests an alteration in the polysaccharide structure of cell walls. This observation potentially reflects altered cellulose and hemicellulose compositions and coincides with the observed inhibition of proliferated shoot growth. This finding highlights the critical role of cell wall polysaccharide synthesis in maintaining the dynamic structure required for proper proliferated shoot development.

Peaks in the 896.46–456.72 cm^−1^ region corresponded to various carbohydrates [63]. Changes in carbohydrate peak profiles following Co treatments indicate alterations in their cellular or cell wall distribution. Investigating the impact of Co stress on macromolecule synthesis is crucial for understanding how plants respond to HM exposure.

## Conclusions

The results indicated that the lowest cobalt concentration (50 µM) promoted the growth of proliferated shoots. Higher cobalt concentrations, however, showed negative impacts on the proliferated shoots’ development, water content, and photosynthetic pigments. Increased cobalt concentrations led to oxidative damage, as shown by higher MDA levels. In response to higher cobalt concentrations, plants increased the production of total antioxidants, ascorbate, phenolic compounds, proline, free amino acids, soluble proteins, and soluble carbohydrates. The positive correlation between these compounds and growth damage suggests that they may be involved in the defensive system against cobalt stress. In addition, FTIR analysis revealed that macromolecules may play a role in chelating cobalt ions. Overall, these findings suggest that *Adhatoda vasica* proliferated shoots have mechanisms to tolerate cobalt toxicity.

## Materials

### Plant tissue culture

Fresh and healthy shoot tips were collected from *Adhatoda vasica* shrubs that grow on the Botany and Microbiology farm at Assiut University, Egypt (27°11′00″N, 31°10′00″E). They were thoroughly rinsed under running tap water for 20–30 min, transferred to a laminar flow hood, and rinsed three times with sterile distilled water. They were immersed in a 10% sodium hypochlorite solution for 10 min and rinsed thoroughly thrice with sterile distilled water. Three sterilized shoot tips were aseptically inoculated into each 195-mL cultivating jar containing 30 mL of solidified and sterilized Murashige and Skoog (MS) medium [64]. The jars were incubated in a growth chamber under controlled conditions [photoperiod (16/8 h’ light/dark), light intensity (30 μmol m^−2^ s^−1^), temperature (25 ± 1 °C), and relative humidity (50–60%)]. After 30 days, shoots that exhibited proliferation (the development of new shoots) were harvested, and their fresh weight (FW) was determined.

The MS medium included 4.4 g L^−1^ of Murashige and Skoog salts, 3% sucrose, 3 mg L^−1^ of 6-benzylaminopurine, and 1 mg L^−1^ of α-naphthalene acetic acid. Various concentrations of cobalt chloride [CoCl_2_·6H_2_O (0, 50, 100, 200, 400, 600, and 1000 µM)] were added to the MS medium. The pH of the culture medium was adjusted to 5.7 before the 3 g L^−1^ gelrite addition. The medium was autoclaved for 15 min at 121 °C, a pressure of 105 kPa, and then cooled to solidify.

After harvesting, the shoots were weighed promptly and frozen immediately in liquid nitrogen for storage at −80 °C. Other shoots were oven-dried at 60 °C for 48 h to determine the dry weight (DW). Total water content was calculated by subtracting the DW from the FW and expressed as grams per shoot tip.

### Percent of damage

After Co stress, the relative decrease in proliferating tissue was used to quantify shoot tip growth inhibition. This was calculated using the following formula [65]:

$$\%\;\mathrm{of}\;\mathrm{growth}\;\mathrm{damage}=100\times\left[\left(\mathrm{DW}\;\mathrm{control}\right)-\left(\mathrm{DW}\;\mathrm{cobalt}-\mathrm{treated}\right)\right]/\;\left(\mathrm{mean}\;\mathrm{DW}\;\mathrm{control}\right)$$

### Photosynthetic pigments

Photosynthetic pigments (chlorophyll *a*, *b*, and carotenoids) were quantified spectrophotometrically [66]. Fresh leaf tissue (0.1 g) was extracted in 5 mL of 95% ethanol at 60 °C until decolorized. The mixture was then diluted with 95% ethanol to make 10 mL. Chlorophyll and carotenoid concentrations were measured spectrophotometrically using Unico UV-2100 and specific formulas.

$$\begin{array}{l}\text{Chl}.\;a=\left(13.36\times\text{A}663\right)-\left(5.19\times\text{A}644\right)\\\text{Chl}.\;b=\left(27.49\times\text{A}644\right)-\left(8.12\times\text{A}663\right)\\\text{Caroteoids}=\left[\left(1000\times\text{A}452\right)-\left(2.13\times\text{chl}.\;a\right)-\left(9.76\times\text{chl}.\;b\right)\right]/209.\end{array}$$

Chlorophyll and carotenoid concentrations were calculated based on fresh weight (mg g^−1^ FW).

### Lipid peroxidation

Lipid peroxidation was assessed by measuring malondialdehyde (MDA) concentration using the thiobarbituric acid (TBA) reaction [67]. Cellular components were extracted by grinding a 0.2 g sample in 5 mL trichloroacetic acid (0.1%; TCA). The resultant mixture was spun at 10,000 rpm for 10 min in a cold environment (4 °C). One mL of the supernatant was combined with 4 mL of TCA (20%) and TBA (0.5%). For 30 min, the mixture was heated to 95 °C. After cooling, the reaction mixture was spun at 10,000 rpm for 15 min. The supernatant’s absorbance was calculated at 532 nm by a spectrophotometer. MDA concentration was calculated as mM g^-1^ fresh weight (FW) and expressed at an extinction coefficient of 155 mM cm^-1^.

### Total antioxidant

Total antioxidant capacity was determined using the phosphomolybdenum method [68]. A 0.5 g sample was ground in 5.0 mL of 100 mM potassium phosphate buffer (pH 7.8) containing 0.1 mM ethylenediamine tetra-acetic acid, disodium salt, and 0.1 g polyvinylpyrrolidone. The resultant mixture was spun at 18,000 rpm for 10 min in a cold environment (4 °C). The supernatant (50 µL) was combined with 3 mL of the reagent solution containing 0.6 M sulfuric acid, 28 mM sodium phosphate, and 4 mM ammonium molybdate. The mixture was incubated in a water bath at 95 °C for 90 min. After cooling, the absorbance was measured at 695 nm using a spectrophotometer, and the total antioxidant was expressed as mg g^−1^ FW.

### Ascorbic acid

Ascorbic acid (AsA) concentration was determined using the Folin-Ciocalteu reagent [69]. After grinding a 0.3 g sample with 2 mL of TCA (5%) at 4 °C for 15 min, the sample was spun at 10,000 rpm. After combining the 200 µL of supernatant with 0.8 mL of 10% TCA, it was incubated in an ice bath for 5 min. The mixture was spun at 3,000 rpm for 5 min. Then, 0.5 mL of the supernatant was diluted with water to a final volume of 2 mL. The diluted solution was mixed with 0.2 mL of 0.2 M Folin-Ciocalteu reagent. Next, the mixture was incubated for 10 min. The absorbance was measured at 760 using a spectrophotometer. A calibration curve was prepared with varying AsA concentrations, and the data were expressed as mg g^-1^ FW.

### Phenolic compounds

Free and cell-wall-bound phenolic concentrations were determined using the Folin-Ciocalteu method [70]. The sample underwent a 90-min extraction at 80 °C with 6 mL of 50% methanol and was spun at 14,000 rpm for 15 min. The pellet was incubated with 2 mL of NaOH solution at room temperature for 24 h, then neutralized with 0.5 mL of HCl and spun at 14,000 rpm for 15 min. Aliquots (50 µL) of both the methanol extract (free phenolics) and the NaOH extract (bound phenolics) were diluted with water to a final volume of 1 mL. The mixture was supplemented with 2.5 mL of sodium carbonate solution and 0.5 mL of 2N Folin-Ciocalteu’s phenol reagent. The mixture was then incubated for 20 min at room temperature, and the absorbance was measured at 725 nm by a spectrophotometer. Phenolic concentrations were expressed relative to a gallic acid standard curve and calculated as mg g^−1^ FW.

### Proline

Proline concentration was measured using the acid-ninhydrin method described by Bates et al. [71]. The sample was crushed and mixed thoroughly with 3 mL of a 3% sulfosalicylic acid solution. This mixture was spun at 10,000 rpm for 10 min. One mL of the supernatant was combined with 2 mL of glacial acetic acid and 2 mL of acid-ninhydrin reagent. The mixture was then shaken with 4 mL of toluene. Absorbance was measured spectrophotometrically at 520 nm. Proline concentration was measured and expressed as mg g^−1^ FW.

### Free amino acids

Free amino acid concentrations were determined using the ninhydrin method with glycine as a standard [72]. An aliquot (200 µL) of the total antioxidant extract was combined with 1 mL of SnCl_2_ reagent. This mixture was heated to 95 °C for 20 min. The mixture was cooled and diluted with 5 mL of ethanol solvent. Absorption was measured spectrophotometrically at 570 nm. Amino acid concentrations were determined and expressed as mg g^−1^ FW.

### Soluble proteins

Soluble protein concentrations were measured using the Folin-Ciocalteu method with bovine serum albumin calibration [73]. An aliquot (50 µL) of the total antioxidant extract was combined with 5 mL of alkaline reagent A and 1 mL of reagent B. Reagent A was 2 g of Na_2_CO_3_ dissolved in 100 mL of 0.1 N NaOH. Reagent B was 0.5 g of CuSO_4_.5H_2_O dissolved in 100 mL of 1% sodium potassium tartrate. The mixture was allowed to stand at room temperature for 10 min and mixed rapidly with 0.5 mL of diluted Folin-Ciocalte’s (1:2 v/v) reagent for 30 min. The absorbance of the mixture was measured at 750 nm by a spectrophotometer. Soluble proteins were expressed as mg g^−1^ FW.

### Soluble carbohydrates

Soluble carbohydrate concentration was measured using the Albalasmeh [74] sulfuric acid-UV method, monitoring absorbance at 315 nm. An aliquot (1 mL) of the total antioxidant extract was quickly mixed with 3 mL of concentrated sulfuric acid and shaken vigorously for 30 s. Soluble carbohydrates were measured at 315 nm and expressed as mg g^−1^ FW.

### Fourier transform infrared

At the Chemistry Department of Assiut University in Egypt, macromolecular alterations were examined by Fourier transform infrared (FTIR) spectroscopy (Nicolet IS 10). A dry, powdery proliferating shoot sample of around 100 μg was combined with dry potassium bromide in a 1:100 w/w ratio. Using a tablet press, the mixture was compacted into homogenous, translucent tablets. FTIR analysis was performed at 4000–400 cm^−1^ with a 4 cm^−1^ resolution.

### Statistical analysis

Experiments were conducted at least two times and four replicates per treatment, each containing 25 jars. Data are presented as the mean ± standard deviation (SD) of four biological replicates. Origin 8.6 and Microsoft Excel 2010 were used to display the FTIR data. For statistical analysis of data, SPSS Statistical Package 26.0 was used to perform an ANOVA (one-way analysis of variance), followed by a Tukey’s posthoc test to identify specific parameter differences (*P* ≤ 0.05). Pearson’s correlation coefficients were calculated and expressed in a heatmap to evaluate the relationships between the mean values of various parameters in *A. vasica* exposed to Co treatments.

## Supplementary Information

Supplementary Material 1.

## Data Availability

Data will be made available on request.

## Abbreviations

AsA: Ascorbic acid
Chl.*a*: Chlorophyll *a*
Chl.*b*: Chlorophyll *b*
Co: Cobalt
DW: Dry weight
FTIR: Fourier transform infrared
FW: Fresh weight
HM: Heavy metal
MDA: Malondialdehyde
MS: Murashige and Skoog medium
ROS: Reactive oxygen species
SD: Standard deviation
TBA: Thiobarbituric acid
TWC: Total water content
TCA: Trichloroacetic acid
