# Supplementary material for: Cobalt-induced oxidative stress and defense responses of Adhatoda vasica proliferated shoots
Source: BMC Plant Biol. 2025 Jan 31;25:132. doi: 10.1186/s12870-024-05915-7 (PMC11783736; doi:10.1186/s12870-024-05915-7)
Supplement: Supplementary file 1 — Supplementary Material 1. [file 12870_2024_5915_MOESM1_ESM.docx]

**Table 1:** Pearson correlation analysis of *Adhatoda vasica* parameters following 30-day of different cobalt concentrations exposure.

|  | **Correlations** | | | | | | | | | | | | | | | | | | | | | | |
| --- | --- | --- | --- | --- | --- | --- | --- | --- | --- | --- | --- | --- | --- | --- | --- | --- | --- | --- | --- | --- | --- | --- | --- |
|  | **% of growth damage** | | **Total water content** | **Chlorophyll a** | **Chlorophyll b** | **Carotenoids** | | **MDA** | | **Proline** | | **Amino acids** | | **Total antioxidants** | | **Ascorbic acid** | | **Free Phenolics** | | **Bound Phenolics** | **Soluble proteins** | | **Soluble carbohydrates** |
| **% of growth damage** |  |  | |  |  |  | |  | |  | |  | | |  |  | |  | |  |  | |  |
| **Total water content** |  |  | |  |  |  | |  | |  | |  | | |  |  | |  | |  |  | |  |
| **Chlorophyll a** |  |  | |  |  |  | |  | |  | |  | | |  |  | |  | |  |  | |  |
| **Chlorophyll b** |  |  | |  |  |  | |  | |  | |  | | |  |  | |  | |  |  | |  |
| **Carotenoids** |  |  | |  |  |  | |  | |  | |  | | |  |  | |  | |  |  | |  |
| **MDA** |  |  | |  |  |  | |  | |  | |  | | |  |  | |  | |  |  | |  |
| **Proline** |  |  | |  |  |  | |  | |  | |  | | |  |  | |  | |  |  | |  |
| **Amino acids** |  |  | |  |  |  | |  | |  | |  | | |  |  | |  | |  |  | |  |
| **Total antioxidants** |  |  | |  |  |  | |  | |  | |  | | |  |  | |  | |  |  | |  |
| **Ascorbic acid** |  |  | |  |  |  | |  | |  | |  | | |  |  | |  | |  |  | |  |
| **Free Phenolics** |  |  | |  |  |  | |  | |  | |  | | |  |  | |  | |  |  | |  |
| **Bound Phenolics** |  |  | |  |  |  | |  | |  | |  | | |  |  | |  | |  |  | |  |
| **Soluble proteins** |  |  | |  |  |  | |  | |  | |  | | |  |  | |  | |  |  | |  |
| **Soluble carbohydrates** |  |  | |  |  |  | |  | |  | |  | | |  |  | |  | |  |  | |  |
| **. Correlation is significant at the 0.01 level (2-tailed). | | | | | | |  | |  | |  |  |  | | | |  | |  |  |  |  | |

**Color key**

| **The positive value** |  |  |  |  |  |  |  |  |  | **The negative value** |
| --- | --- | --- | --- | --- | --- | --- | --- | --- | --- | --- |
